# Clinical study on bronchial artery chemoembolization for unresectable non-small cell lung cancer

**DOI:** 10.3389/fonc.2025.1591752

**Published:** 2025-09-03

**Authors:** Fenxiang Zhang, Yujin Liu

**Affiliations:** Department of Interventional Oncology, Yueyang Hospital of Integrated Traditional Chinese and Western Medicine, Shanghai University of Traditional Chinese Medicine, Shanghai, China

**Keywords:** bronchial arterial chemoembolization, chemotherapy, non-small cell lung cancer, progress free survival, overall survival

## Abstract

**Objective:**

To investigate the efficacy and safety of bronchial artery chemoembolization (BACE) for the treatment of inoperable non-small cell lung cancer (NSCLC).

**Methods:**

A retrospective review was conducted of 112 patients with NSCLC who received BACE treatment and 120 patients who underwent systemic chemotherapy in our center over the past 10 years. The progression-free survival (PFS), overall survival (OS), objective response rate (ORR), quality of life, and adverse events were compared between the two groups.

**Results:**

The complete response (CR), partial response (PR), stable disease (SD), and progressive disease (PD) rates in the BACE group were 7.14%, 39.29%, 33.04%, and 20.54%, respectively, while those rates in the chemotherapy group were 7.5%, 20.83%, 55.0%, and 16.67%. The ORR in the BACE group was significantly higher than in the chemotherapy group (46.43% vs. 28.33%, *P* = 0.007). The median PFS was significantly longer in the BACE group (17 months vs. 11 months, *P* = 0.035) than that of chemotherapy group, as was the median OS (19.5 months vs. 13 months, *P* = 0.044). The BACE group also showed significantly better results in cough relief (57.0% vs. 41.7%, *P* = 0.028) and hemoptysis relief (70.3% vs. 47.1%, *P* = 0.001) compared to the chemotherapy group. The 1-year, 3-year, and 5-year survival rates for the BACE group were 68.0%, 29.1%, and 15.5%, respectively, compared to 49.5%, 15.9%, and 7.5% those of the chemotherapy group, with statistically significant differences (*P* < 0.05). The BACE group exhibited better tolerability and higher safety, with a lower incidence and severity of adverse events, particularly fatigue, nausea/vomiting, and myelosuppression, which showed statistically significant differences (*P* < 0.05).

**Conclusion:**

BACE treatment for inoperable NSCLC offers better clinical outcomes compared to systemic chemotherapy and is safe and well-tolerated, with no severe adverse events. This approach warrants further prospective randomized controlled trials.

## 1 Introduction

Non-small cell lung cancer (NSCLC) is a common and highly fatal malignancy, with both incidence and mortality rates showing an upward trend globally. According to statistics, approximately 2.2 million new cases of lung cancer were reported worldwide in 2022, of which NSCLC accounted for approximately 80%-85% of the cases (1). The treatment of NSCLC typically includes surgery, chemotherapy, radiotherapy, and targeted drug therapy. However, due to the subtle early symptoms of NSCLC, many patients are diagnosed at an advanced stage (2, 3), and the low rate of curative surgery renders surgical resection no longer a viable treatment option (4). While systemic chemotherapy can delay disease progression and improve symptoms to some extent, its effectiveness is limited and often accompanied by severe adverse effects. Molecular targeting and immunotherapy have represented major breakthroughs in lung cancer treatment over the past decade, particularly for NSCLC patients with specific gene mutations or high immune expression (5). These approaches have become first-line treatment options, but patients may develop resistance, compromising treatment efficacy. Bronchial artery infusion chemotherapy (BAI) and bronchial artery chemoembolization (BACE) have been clinically applied for a considerable time but have yet to become standard treatment options. Our center has implemented BACE treatment for lung cancer with successful clinical outcomes (11, 14, 20). This retrospective study compares the efficacy and safety of BACE treatment for inoperable NSCLC with that of systemic chemotherapy.

## 2 Methods

### 2.1 Case information

This study presents a systematic review of 112 cases of lung cancer treated with BACE and 120 cases treated with systemic chemotherapy at the Yueyang Integrated Traditional Chinese and Western Medicine Hospital of Shanghai University of Traditional Chinese Medicine over a 10-year period from 2014 to 2023, comparing their progression-free survival (PFS), overall survival (OS), objective response rate (ORR), quality of life improvement, and adverse events. This study was approved by the Hospital Ethics Committee (Approval No: 2024-178).

#### 2.1.1 Inclusion criteria

1. Histologically or cytologically confirmed diagnosis of non-small cell lung cancer (NSCLC);
2. Imaging assessment for those with unresectable tumors or patients who are unsuitable for surgery, radiotherapy, local ablation, particle implantation, molecular targeted therapy, or immunotherapy, etc;
3. Presence of at least one measurable lesion;
4. Receipt of at least two cycles of systemic chemotherapy or BACE treatment;
5. Consistent chemotherapy regimens (adenocarcinoma: pemetrexed + platinum-based chemotherapy, squamous cell carcinoma: taxane-based chemotherapy + platinum-based chemotherapy).

#### 2.1.2 Exclusion criteria

1. Presence of two or more primary malignant tumors in different locations;
2. Death from non-lung cancer-related causes;
3. Concurrent receipt of other active medical interventions, such as surgery, radiotherapy, or targeted immunotherapy, within two cycles of systemic chemotherapy or BACE treatment.

### 2.2 Treatment methods

#### 2.2.1 BACE treatment

The procedure was performed under sterile conditions with local anesthesia. Using the Seldinger technique, a femoral artery puncture was made, followed by the introduction of a 5-F femoral sheath. A 5F “MIK,” RLG, or 5F “Cobra” catheter was then used for catheterization. Under fluoroscopic guidance, the guidewire and catheter were advanced through the femoral artery to the thoracic aorta. After catheterizing the relevant target artery, a microcatheter was selectively advanced into the bronchial artery, intercostal arteries, subdiaphragmatic arteries, and other target vessels such as the internal thoracic artery, with angiography confirmation at each step. In some cases, cone-beam CT was used to confirm the integrity of the tumor’s blood supply and to exclude blood supply from the pulmonary arteries.

Two types of microcatheters were used during the procedure. One was the Stride microcatheter (Asahi STD125-26S), with an outer diameter of 2.6Fr (0.88mm) at the tip and an inner guidewire of 0.018Fr (0.46mm), which could be bent into a J-shape as needed. The other was the Merit Maestro microcatheter (Merit Medical System, 29MC29130ST), with an outer diameter of 2.9Fr (0.96mm) at the tip, shaped like a Swan-neck, and an internal guiding wire of Merit Medical Tenor (TNR2811), with an outer diameter of 0.018Fr (0.46mm). The straight-tip guidewire could also be bent into a J-shape as needed. Digital subtraction angiography (DSA) was used to confirm the blood supply to the tumor via the target arteries and to exclude abnormal shunts to the spinal cord or brain.

During the surgery, paclitaxel (60–80 mg/m²) and cisplatin (30–40 mg/m²) or carboplatin (dose calculated based on AUC = 5) are diluted to 50–100 ml and slowly injected into the tumor area using a 0.9% saline solution. Each drug is administered for at least 20 minutes. Subsequently, absorbable gelatin sponge particles of 150-350-560 μm are used to embolize the distal target artery to block the blood supply to the tumor while preserving the main trunk of the target artery. After the procedure, the catheter is removed, pressure is applied to the puncture site to stop bleeding, and the site is bandaged. The patient must remain in bed with the puncture site immobilized for 10 hours. Vital signs and complications are monitored throughout the procedure. Symptomatic treatment is provided based on the patient’s response.

The treatment schedule involves chemotherapy embolization every 3–4 weeks for a total of 3–6 cycles, with the specific regimen adjusted based on the patient’s condition and treatment response. Blood cell counts, liver and kidney function tests, tumor markers, etc., are rechecked every 3–4 weeks. Contrast-enhanced CT scans are performed every 3 months to assess tumor size and changes in blood supply. If necessary, ultrasound, MRI, and bone scans are used to evaluate liver, brain, and bone metastasis.

#### 2.2.2 Systemic chemotherapy

The systemic chemotherapy regimens were tailored based on the type of cancer. For adenocarcinoma patients, pemetrexed (500 mg/m²) combined with a platinum-based drug (cisplatin 75–100 mg/m² or carboplatin with AUC 5–6) was used. For squamous cell carcinoma patients, taxane-based drugs (paclitaxel 135–175 mg/m² or docetaxel 75 mg/m²) were combined with a platinum-based agent. According to the retrospective analysis, these treatment protocols generally followed a 3-week cycle, after which a rest period was allowed to monitor side effects and provide symptomatic treatment as necessary. Imaging evaluations were performed after every 2–3 cycles to assess the treatment effect and adjust drug doses and regimens accordingly. Typically, 3–6 cycles of chemotherapy were administered. At the conclusion of the cycles, a comprehensive evaluation was made to decide whether maintenance therapy or additional cycles were necessary. All treatment regimens and adjustments were personalized based on the patient’s individual condition and clinical response to ensure maximum therapeutic efficacy with minimal side effects. These treatment protocols and cycles were based on the retrospective analysis of completed treatments.

### 2.3 Evaluation metrics

PFS (Progression-Free Survival): The time from the start of treatment until disease progression or death occurs.

OS (Overall Survival): The time from the start of treatment until death from any cause.

1. Evaluation Criteria: RECIST 1.1:The evaluation criteria will record the following parameters for each group:
  Complete Response (CR): The total disappearance of all target lesions.
  Progressive Disease (PD): At least a 20% increase in the sum of the longest diameters of target lesions, or the appearance of new lesions.
  Partial Response (PR): A decrease of at least 30% in the sum of the longest diameters of target lesions.
  Stable Disease (SD): Neither sufficient shrinkage to qualify for PR nor sufficient increase to qualify for PD.
  Objective Response Rate (ORR): The proportion of patients who achieve either CR or PR.
2. Disease Control Rate (DCR): The proportion of patients who achieve CR, PR, or SD.
3. Adverse Events (AE): Record any adverse events that occur during the treatment process.

### 2.4 Statistical methods

Data entry and database construction will be performed using SPSS version 27.0 statistical software. The comparison of baseline characteristics between groups will be conducted using the chi-square test. Survival analysis will use the life table method to calculate survival rates, and survival curves will be plotted. The Log-rank test will be used to analyze factors that may influence survival time. The significance level is set at α = 0.05.

## 3 Results

### 3.1 Baseline characteristics of patients

A total of 232 patients with inoperable non-small cell lung cancer (NSCLC) were included in the study, with 112 patients in the BACE group and 120 patients in the chemotherapy group. No statistically significant differences were observed between the two groups regarding basic clinical characteristics, including gender, age, pathological classification, TNM staging, distant metastasis, primary tumor site, main symptoms before treatment, and subsequent treatments (**Table 1**). The median follow-up time was 11 months (follow-up data was collected until September 30, 2024). At the end of the follow-up period, 157 patients had died, with 77 deaths in the BACE group and 80 deaths in the chemotherapy group. In addition, 9 deaths were recorded due to non-cancer-related causes (such as cardiovascular accidents, other accidents, etc.), with 4 from the BACE group and 5 from the chemotherapy group. According to the exclusion criteria, patients who died from non-cancer-related causes were excluded from the final data analysis. A total of 112 patients were included in the BACE group, with a loss-to-follow-up rate of 8.04%, while 120 patients were included in the chemotherapy group, with a loss-to-follow-up rate of 10.83%.

**Table 1:** Baseline clinical characteristics of patients.

Characteristic	BACE group (N = 112)	Chemotherapy group (N = 120)	X²/t value	*P* value
Sex				
Male	76	90	0.007	0.934
Female	36	30		
Age			0.816	0.416
<65	60	53		
65-74	40	56		
≥75	12	11		
Pathological classification				
Squamous Cell Carcinoma	1	31	0.431	0.512
Adenocarcinoma	71	89		
TNM stage				
IIIb	46	58	0.761	0.383
IVa	39	44		
IVb	27	18		
Primary tumor location				
Central-type	25	48	0.665	0.415
Peripheral-type	87	72		
Main symptoms				
Cough	72	83	0.645	0.886
Hemoptysis	37	34		
Dyspnea	69	75		
Chest pain	25	28		
Subsequent treatment				
Radiotherapy	20	25	1.81	0.614
Ablation	15	10		
Surgery	5	4		
Best Supportive Care	39	43		
KPS score before treatment				
60-70	34	26	2.200	0.334
71-80	48	59		
>80	30	35		

### 3.2 Efficacy evaluation

The results indicated that the BACE group demonstrated statistically significant differences compared to the chemotherapy group in terms of partial response (PR), stable disease (SD), and objective response rate (ORR), with P-values of 0.003, 0.001, and 0.007, respectively. The BACE group showed significantly better outcomes, suggesting that BACE may be more effective in reducing tumor burden in the short term. No significant differences were observed between the two groups regarding complete response (CR), progressive disease (PD), and disease control rate (DCR) (**Table 2**).

**Table 2:** Tumor response rate in both groups.

Group	CR	PR	SD	PD	ORR (CR+PR)	DCR (CR+PR+SD)
BACE Group	8 (7.14%)	44 (32.29%)	37 (33.04%)	23 (20.54%)	52 (46.43%)	89 (79.46%)
Chemotherapy Group	9 (7.5%)	25 (20.83%)	66 (55.0%)	20 (16.67%)	34 (28.33%)	100(83.33%)
*P*-value	1	0.003	0.001	0.556	0.007	0.556

The results indicate that the BACE group exhibited statistically significant differences in the relief rates for cough and hemoptysis compared to the chemotherapy group, with *P*-values of 0.028 and 0.001, respectively. The BACE group showed significantly better outcomes. The relief rate for dyspnea showed a smaller difference between the two groups, with the BACE group achieving 71.9% and the chemotherapy group 60.0% (*P* = 0.077), demonstrating better efficacy in the BACE group. Similarly, the difference in the relief rate for chest pain between the two groups was modest, with the BACE group at 68.0% and the chemotherapy group at 57.1% (*P* = 0.115) (**Table 3**).

**Table 3:** Comparison of major symptoms before and after treatment in both groups.

Symptom	BACE group (Pre-treatment)	BACE group (Post-treatment)	Chemotherapy group (Pre-treatment)	Chemotherapy group (Post-treatment)	Relief rate (BACE Group)	Relief rate (Chemotherapy Group)	*P*-value
Cough	72 (69.9%)	25 (24.3%)	83 (77.6%)	34 (31.8%)	57.0%	41.7%	0.028
Hemoptysis	37 (35.9%)	12 (11.7%)	34 (31.8%)	18 (16.8%)	70.3%	47.1%	0.001
Dyspnea	69 (67.0%)	20 (19.4%)	75 (70.1%)	30 (28.0%)	71.9%	60.0%	0.077
Chest Pain	25 (24.3%)	8 (7.8%)	28 (26.2%)	12 (11.2%)	68.0%	57.1%	0.115

As shown in **Table 4**, the average KPS score for the BACE group before treatment was 65.4, and it improved to 80.1 after treatment, with a mean change of +14.7. In the chemotherapy group, the average KPS score before treatment was 66.1, which increased to 75.3 after treatment, with a mean change of +9.2. Between-group comparison revealed a statistically significant difference (*P* = 0.001), with the BACE group demonstrating a significantly greater improvement in KPS scores compared to the chemotherapy group.

**Table 4:** Comparison of KPS scores before and after treatment in both groups.

Group	Mean KPS (Pre-treatment)	Mean KPS (Post-treatment)	t-value	*P*-value
BACE Group	65.4 ± 10.3	80.1 ± 9.4	15.13	0.001
Chemotherapy Group	66.1 ± 9.8	75.3 ± 8.1	10.59	0.001
Between Groups	0.614	0.001	4.50	0.001

**Figure 1:**
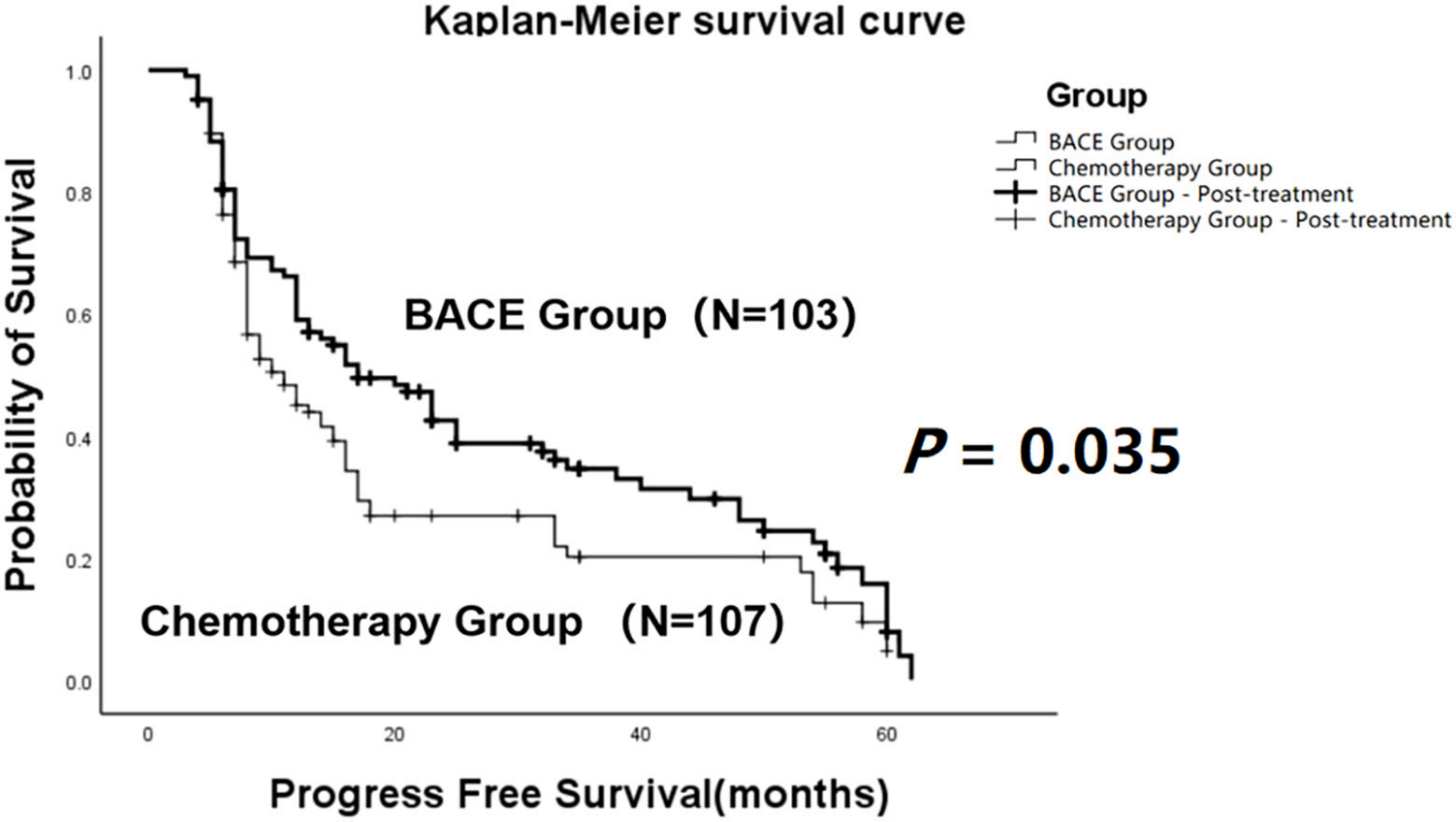
Kaplan-Meier curves comparing progression-free survival (PFS) between the BACE group and the chemotherapy group. A statistically significant difference in PFS was observed between the two groups (*P* = 0.035, Log-rank test).

The comparison demonstrated that the mean progression-free survival (PFS) in the BACE group (N = 103) was 27.29 ± 2.26 months, with a median PFS of 17 months. In the Chemotherapy group (N = 107), the mean PFS was 20.67 ± 2.11 months, and the median PFS was 11 months. Both the mean and median PFS in the BACE group were higher than those in the chemotherapy group. Statistical analysis using the Log-rank (Mantel-Cox) test showed χ² = 4.466 and *P* = 0.035, indicating a statistically significant difference (**Figure 1**).

**Figure 2:**
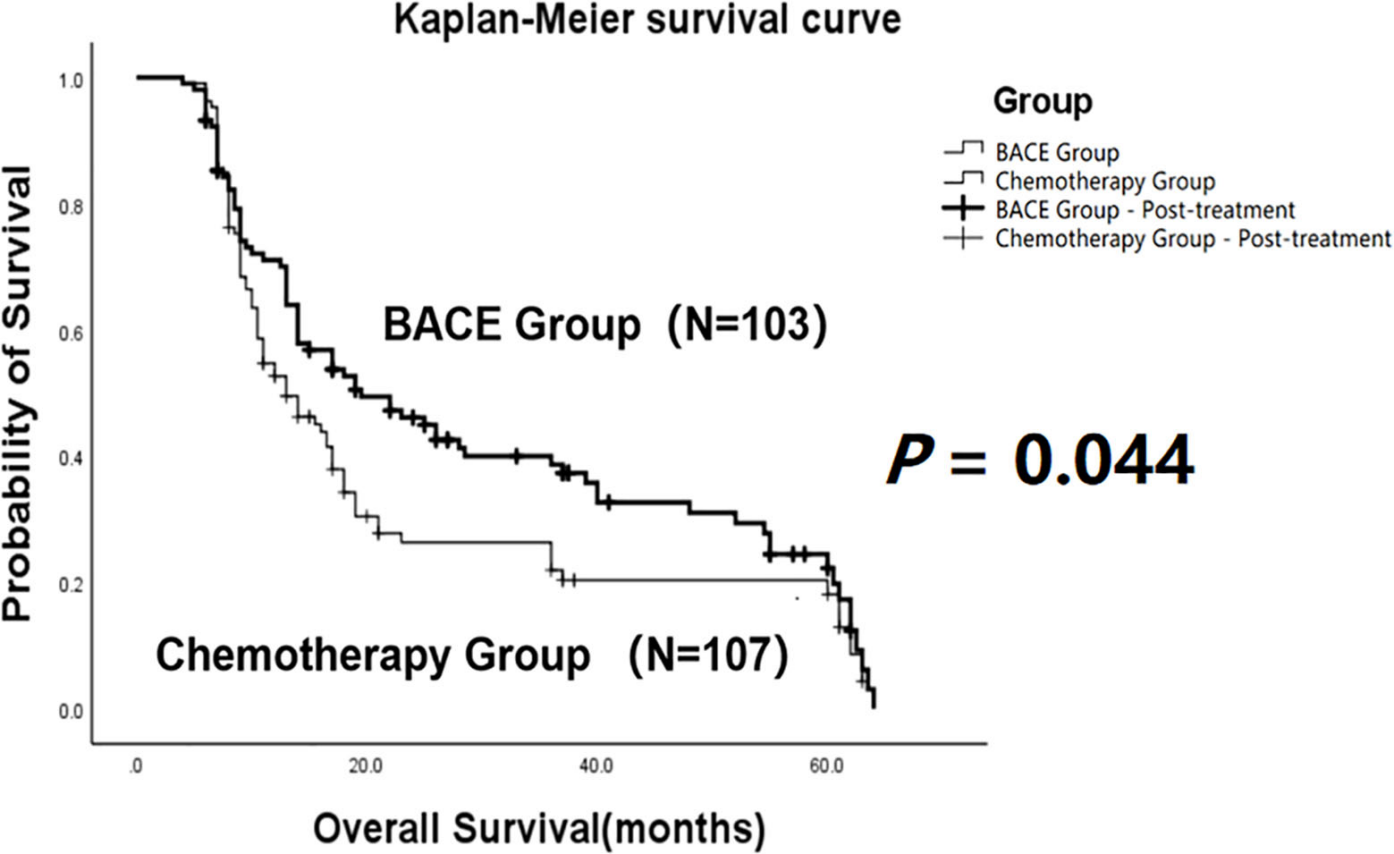
Kaplan-Meier curves comparing overall survival (OS) between the BACE group and the chemotherapy group. A statistically significant difference in OS was observed between the two groups (*P* = 0.044, Log-rank test).

The comparison showed that the mean overall survival (OS) in the BACE group (N = 103) was 30.22 ± 2.33 months, with a median survival time of 19.5 months. In contrast, the mean OS in the chemotherapy group (N = 107) was 23.36 ± 2.22 months, with a median survival time of 13 months. Both the mean OS and median OS in the BACE group were significantly higher than those in the chemotherapy group. Statistical analysis using the Log-rank (Mantel-Cox) test showed χ² = 4.056 and *P* = 0.044, indicating a statistically significant difference (**Figure 2**).

The 1-year, 3-year, and 5-year survival rates for the BACE group and chemotherapy group were 68.0%, 29.1%, and 15.5% versus 49.5%, 15.9%, and 7.5%, respectively. The survival rates at each time point were consistently higher in the BACE group compared to the chemotherapy group. The differences in 1-year survival and 3-year survival rates were statistically significant, with *P*-values of 0.0102 and 0.0327, respectively. However, the difference in the 5-year survival rate was not statistically significant (*P* > 0.05).

### 3.3 Safety evaluation

There was a significant difference in adverse events between the BACE and Chemotherapy groups. The incidence of grade 3–4 adverse events, including fatigue, nausea/vomiting, and myelosuppression, was significantly lower in the BACE group compared to the chemotherapy group, with rates of 1.94%, 2.91%, and 4.86%, respectively, compared to 14.02%, 12.15%, and 15.89% in the chemotherapy group. These differences were statistically significant (*P*-values of 0.003, 0.023, and 0.017, respectively). No significant difference was observed in the incidence of chest pain between the two groups. The incidence of grade 3–4 adverse events in the BACE and Chemotherapy groups was 5.83% and 2.91%, and 5.61% and 7.48%, respectively, with no statistically significant difference (*P*-values of 1.0 and 0.240, respectively) (see **Table 5**).

**Table 5:** Comparison of the incidence of adverse events between the two groups.

Adverse event	Group	Grade 1 (Mild)	Grade 2 (Moderate)	Grade 3 (Severe)	Grade 4 (Very Severe)	Incidence of Grade 3 + 4 (%)	*P*-value
Fatigue	BACE Group	8	7	2	0	1.94%	0.003
	Chemotherapy Group	20	12	9	6	14.02%	
Gastrointestinal Reactions	BACE Group	4	3	3	0	2.91%	0.023
	Chemotherapy Group	15	9	7	6	12.15%	
Bone Marrow Suppression	BACE Group	7	6	5	0	4.86%	0.017
	Chemotherapy Group	16	12	8	9	15.89%	

Additionally, the BACE group exhibited mild skin bruising and hematoma around the puncture site in approximately 4% (4/103) of cases, which resolved spontaneously within 1–2 months. No major bleeding or serious complications, such as paraplegia, were observed.

## 4 Discussion

The anatomical basis for BACE (bronchial artery chemoembolization) in the treatment of lung cancer is that the bronchial arteries, rather than the pulmonary arteries, supply blood to lung tumors (7–9). During tumor growth, the bronchial arteries typically undergo vascular remodeling, leading to increased blood flow. By precisely injecting chemotherapy drugs through a catheter and performing embolization, the concentration of chemotherapy agents in the tumor region can be enhanced, while blocking the tumor’s blood supply arteries, limiting blood flow to the tumor, and increasing the retention time of the drugs locally, thereby improving therapeutic efficacy. Some authors have raised concerns about the difficulty of catheter insertion and the risk of ectopic embolization via the bronchial artery, and have instead used pulmonary artery infusion chemotherapy (10), although no definitive pulmonary arterial blood supply has been identified. In our center, BACE and bronchial artery embolization (BAE) for lung cancer and massive hemoptysis have been performed over a thousand times with successful catheterization and achievement of therapeutic goals, without serious complications such as spinal cord injury (6, 11, 20). In the BACE group, some patients experienced transient coughing and aggravated chest pain during the procedure, which generally resolved within a few hours post-operation and was related to drug stimulation. Therefore, the choice of drugs for BACE avoids those with strong local irritative effects, such as gemcitabine and vincristine, or further dilutes the infusion. Pre-existing symptoms like coughing and chest pain usually resolve after the procedure. With the continuous advancement of catheter techniques, vascular imaging, and microcatheter technology, evidence supporting the predominance of bronchial artery blood supply to lung cancer is increasingly robust. The success rate of BACE procedures is steadily increasing, and compared to traditional pulmonary artery infusion, BACE more effectively enhances drug concentration in the tumor area, reduces damage to normal lung tissue, and is expected to become an important option for the treatment of advanced lung cancer. Our center’s clinical research findings on lung cancer blood supply will be published in future studies.

**Figure 3:**
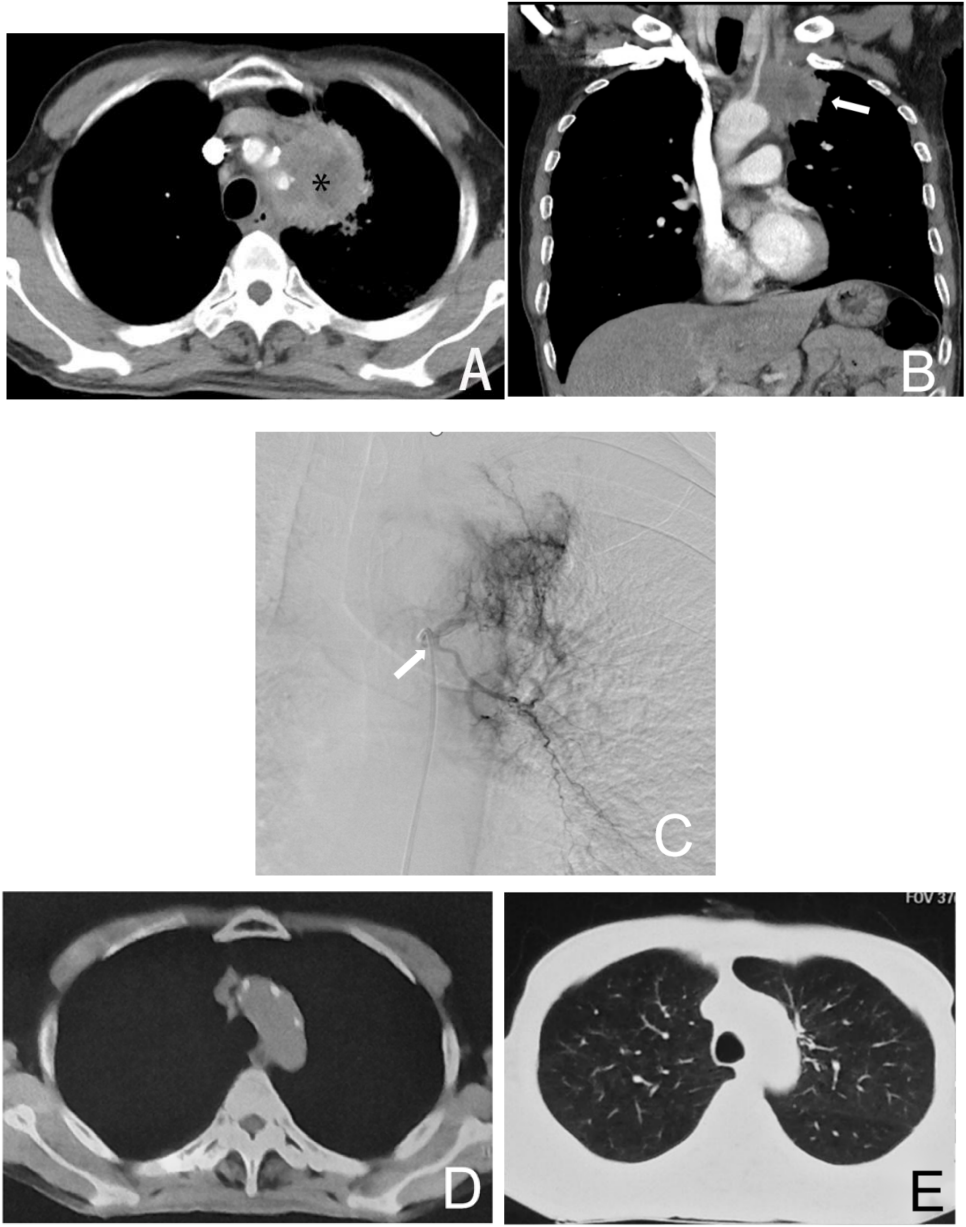
Male, 61 years old, diagnosed with left lung adenocarcinoma through a puncture biopsy, without driver mutations (no molecularly targeted therapy). Figure **(A)** shows the CT axial image of the chest, where the left upper lung cancer surrounds a major blood vessel, making it inoperable (*). Figure **(B)** shows the coronal image of the patient, indicating the left lung tumor (→). Figure **(C)** is a left bronchial arteriogram showing that the left lung tumor was supplied by the left bronchial artery with a rich blood supply (→). Figures **(D, E)** demonstrate that after four courses of BACE treatment, the tumor has disappeared, with the treatment achieving CR.

This study compares the relative advantages and safety of BACE versus systemic chemotherapy in patients with non-small cell lung cancer (NSCLC) (25). By directly targeting the tumor’s blood supply arteries, BACE is able to more effectively control local tumor growth, making it particularly suitable for patients with inoperable lung cancer (**Figure 3**) (6, 12–15). The advantage of BACE in partial remission rate and objective response rate may be attributed to its localized high concentration chemotherapy agents directly targeting the tumor area. The BACE group showed significantly higher rates of relief in cough and hemoptysis compared to the chemotherapy group (*P* = 0.028 and 0.001, respectively), and also exhibited better relief of dyspnea (71.9% vs. 60.0%, *P* = 0.077). Overall, BACE showed satisfactory improvement in major symptoms in patients.

BACE treatment demonstrated a certain advantage in short-term survival rates, but long-term effects need further research. Compared to traditional systemic chemotherapy, BACE delivers drugs locally, reducing their distribution throughout the body (16, 17), thus minimizing systemic side effects such as fatigue, gastrointestinal reactions, and bone marrow suppression, which enhances the patient’s quality of life (13, 18). Furthermore, BACE is a minimally invasive technique that can be repeated (3, 16, 19), making it a feasible treatment for patients who cannot undergo surgery or require long-term control. BACE can also be combined with other treatment modalities (such as radiotherapy and immunotherapy) to provide a multifaceted approach (18, 20), improving the overall therapeutic effect. Using precise imaging techniques (15, 17), BACE targets the tumor’s blood supply arteries for treatment (18), reducing damage to surrounding normal tissues. Through these mechanisms and advantages, BACE has demonstrated satisfactory efficacy and good tolerability in treating inoperable NSCLC, making it an important therapeutic option.

BACE has shown significant efficacy and safety in several studies. In a 2022 study by Haili Cao (21), chemotherapy combined with BACE treatment improved the overall response rate and disease control rate in lung cancer patients while reducing the incidence of severe adverse reactions. A study by Bin Shang (22) in 2020 demonstrated the effectiveness of BACE in lung cancer treatment, with survival rates improving over time. In a 2022 study by Hori A (23), BACE treatment showed high response rates in patients with advanced or recurrent lung cancer, particularly in adenocarcinoma patients who had longer survival. Sheng Xu (24) in 2022 reported that BACE was effective in small cell lung cancer patients with high safety. Our preliminary study (6) analyzing 30 cases of refractory central lung cancer with atelectasis showed that BACE treatment significantly improved the patients’ performance status, with the Karnofsky Performance Status (KPS) score increasing from 68.3 to 82.0. Among the cases treated, 20 had complete (CR) or partial response (PR), 9 had stable disease (SD), and only 1 had progressive disease (PD). A report by our center’s Huang Kunlin (14) described a 49-year-old woman with advanced lung cancer who achieved a CR after BACE treatment, which lasted for 8 years. This patient has now been living healthily for over 12 years.

BACE offers better clinical benefits than systemic chemotherapy in treating inoperable NSCLC, with no serious adverse events, particularly providing rapid relief from cough and hemoptysis. Gastrointestinal reactions, bone marrow suppression, and fatigue are less severe, making it worthy of further prospective randomized controlled studies (26).

## Data Availability

The raw data supporting the conclusions of this article will be made available by the authors, without undue reservation.
